# When Tuberculosis Defies Appearances: The Tale of a Deceptive Abdominal Mass on Imaging

**DOI:** 10.7759/cureus.56686

**Published:** 2024-03-22

**Authors:** Hassnae Tkak, Amal Hamami, Aziza Elouali, Zaynab Idri, Nadir Miry, Houssain Benhaddou, Amal Bennani, Imane Kamaoui, Abdeladim Babakhouya, Maria Rkain

**Affiliations:** 1 Department of Pediatrics, Mohammed VI University Hospital, Faculty of Medicine and Pharmacy, Mohamed I University, Oujda, MAR; 2 Department of Pediatric Surgery, Mohammed VI University Hospital, Faculty of Medicine and Pharmacy, Mohamed I University, Oujda, MAR; 3 Department of Anatomopathology, Mohammed VI University Hospital, Faculty of Medicine and Pharmacy, Mohamed I University, Oujda, MAR; 4 Department of Radiology, Mohammed VI University Hospital, Faculty of Medicine and Pharmacy, Mohamed I University, Oujda, MAR

**Keywords:** computed tomography, pseudotumor, tuberculosis, abdomen, mass

## Abstract

Tuberculosis poses a significant public health challenge, especially in highly endemic countries. Rarely, it appears as an abdominal mass resembling a malignant abdominal tumor and can be misleading on imaging, so early diagnosis remains a challenge, and confirmation may require invasive examinations such as laparotomy. The most characteristic radiological appearance is that of a solid, hypervascular, or peripherally enhancing mass with a hypodense center. We present a case of retroperitoneal tuberculosis that simulated a teratoma on imaging. This case highlights the diagnosis difficulties even in endemic countries, despite advances in imaging techniques such as ultrasound and computed tomography.

## Introduction

Tuberculosis is a curable infectious disease that poses a major public health problem. Abdominal localization is relatively common [1]. Pseudotumoral forms of tuberculosis are rare, regardless of location. It is difficult to diagnose and is often diagnosed late, as it can mimic the clinical, radiological, and endoscopic findings of many pathologies [1,2]. Its clinical presentation is often non-specific, and palpation of an abdominal mass may erroneously point to a malignant tumor pathology [3].

## Case presentation

A boy aged four years and nine months had no notable medical history, and there was no recent occurrence of tuberculosis within his family. His admission was prompted by recurring abdominal pain over six months, accompanied by an absence of fever and a noticeable decline in general well-being, as evidenced by asthenia, anorexia, and a weight loss of 3 kg. Upon clinical examination, the child presented in a relatively stable condition, both hemodynamically and respiratorily, and was apyretic. A palpable and tender periumbilical abdominal mass was identified during the abdominal examination.

**Figure 1:**
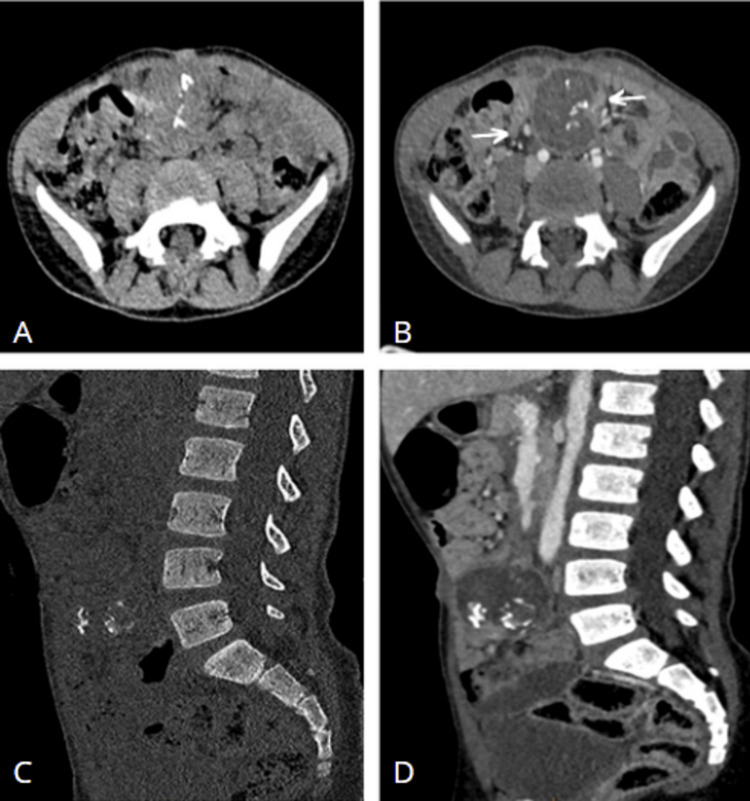
Axial (A+B) and sagittal (C+D) CT scans before and after injection of contrast, showing a mesenteric mass with a hyperdense center, the site of intratumoral calcifications and surrounded by a wall that enhances after injection with peritumoral adenomegaly (arrow).

Further investigations through abdominal ultrasound revealed a sizable retroperitoneal tissue mass. Subsequent abdominal CT scans, conducted before and after contrast injection, unveiled a well-defined, voluminous mesenteric mass measuring 36×40 mm. The mass exhibited distinctive features, including polycyclic contours, an unaltered hypodense center after contrast injection, a few enhanced fine partitions, and vermicular calcifications reminiscent of teeth, indicative of a possible teratoma. This presentation was associated with peritumoral adenomegaly, as illustrated in Figure 1. The chest X-ray yielded normal results.

**Figure 2:**
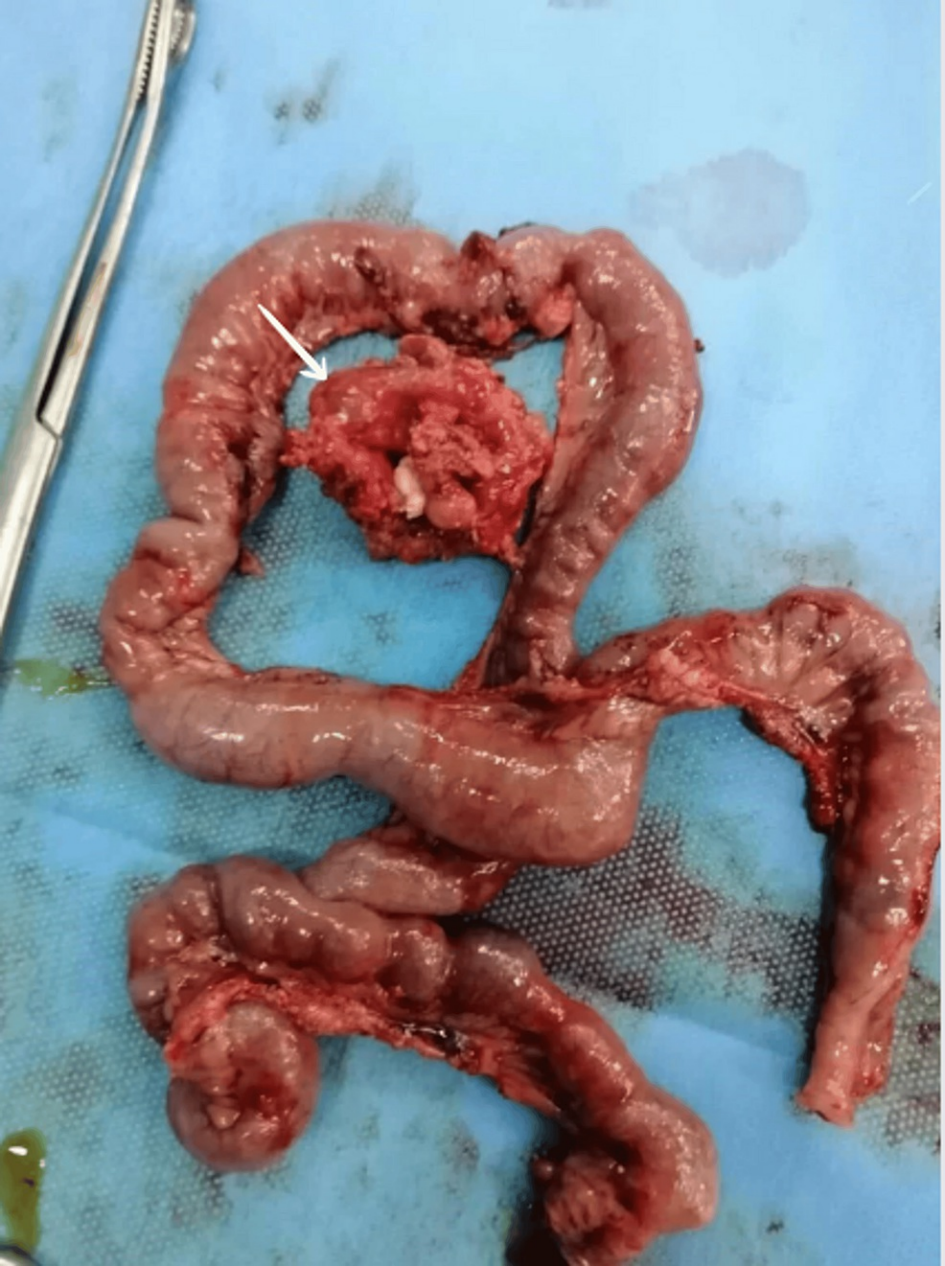
The resected mass (arrow) and the adherent bowel.

Biological assessments showed no signs of an inflammatory syndrome. Urinary catecholamine metabolites, plasma beta-HCG, and alpha-fetoprotein levels were within normal ranges. A laparoscopic examination revealed a retroperitoneal mass firmly adherent to the mesentery, laterally positioned to the right and anterior to the primitive iliac artery. The mass was removed with part of the bowels through laparotomy, as depicted in Figure 2.

**Figure 3:**
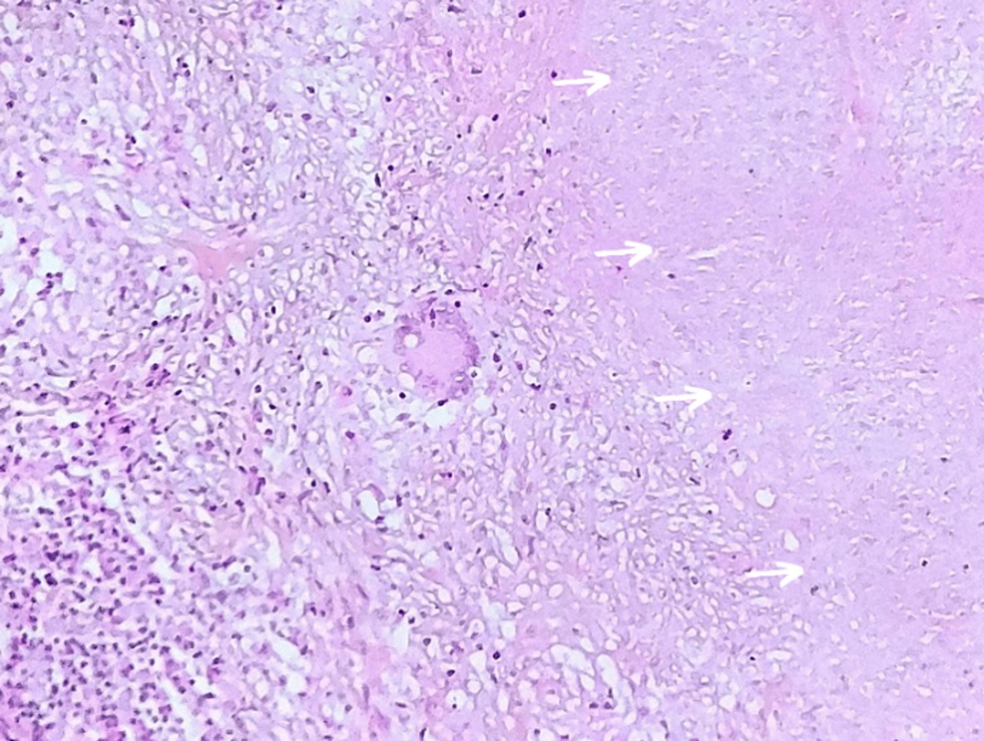
Photomicrograph of the lesion shows a granulomatous reaction characterized by multiple epithelioid cells and Langhans-type multinucleated giant cells. Additionally, a large area of caseous necrosis (arrow) is observed (2: HES, ×400).

The anatomopathological analysis confirmed the presence of an epithelioid and gigantocellular granulomatous lesion with suppurative necrosis and secondary calcification, as shown in Figure 3. Based on the comprehensive evaluation of clinical, radiological, and histological findings, the diagnosis of pseudotumoral abdominal tuberculosis was established. Sputum bacillus (BK) and tuberculin intradermoreaction (TIA) tests were conducted, yielding negative results. The child underwent a six-month course of anti-tuberculosis drugs, resulting in a favorable outcome both clinical and radiological, and no signs of mass in other areas were detected.

## Discussion

The global increase in tuberculosis prevalence can be attributed in part to factors such as immunosuppression linked to human immunodeficiency virus infection, socioeconomic challenges, and population migration [4]. In Morocco, extrapulmonary localizations constitute 15-30% of reported tuberculosis cases, occurring most frequently in lymph nodes and genitourinary, osteoarticular, and neuromeningeal sites [4,5]. Abdominal localization, a relatively common extrapulmonary form, accounts for 5-10% of all cases [1]. The pseudotumoral manifestation of abdominal tuberculosis is a rare clinical-radiological feature, with its frequency challenging to assess but described in only 5% of cases [1,4,5].

Tuberculosis can impact any organ in the abdominal region, yet its clinical and radiological presentation lacks specificity, often leading to undetected diagnoses even in highly endemic regions [1]. Lymph node involvement represents the most common manifestation, with other sites including the digestive tract, peritoneum, liver, and spleen [3,6]. Diagnosis typically occurs late due to clinical and radiological similarities with abdominal tumor pathologies, often necessitating laparotomy in cases of persistent diagnostic uncertainty [4].

Symptoms vary widely and are often misleading, with abdominal distension, pain, and weight loss identified as the primary clinical signs of abdominal tuberculosis in children [7]. An abdominal mass, an exceptional finding, may be erroneously linked to tumoral pathology, especially in the context of altered general condition (AEG) [1,3], posing a challenge for early diagnosis. Clinicians should be vigilant about this localization, considering it in the presence of tumor-like abdominal lesions or when a child exhibits unexplained abdominal complaints, fever, and weight loss, particularly in endemic areas [3,7].

Complementary examinations have limited utility in abdominal tuberculosis unless there is a strong suspicion of the diagnosis [8]. Although abdominal tuberculosis lacks specific radiological signs, imaging techniques play a crucial role in early detection, especially in pseudotumoral forms. CT proves superior to ultrasound, particularly in pseudotumoral forms originating from lymph nodes. The characteristic radiological appearance involves a hypodense formation enhancing peripherally after the injection of contrast medium, indicative of central caseous necrosis with a peripheral inflammatory reaction. While this appearance is specific, it is not pathognomonic for pseudotumoral tuberculosis and is observed in 40-60% of cases [3,9]. In endemic countries, the diagnosis should be considered in the presence of any solid mass exhibiting hypervascularity or peripheral enhancement with a hypodense center. Magnetic resonance imaging contributions in this abdominal location are non-specific, displaying lesions in hyposignal T1 with a variable T2 signal. In the presence of highly suggestive imaging, an etiological work-up should be initiated to screen for other localizations supporting the diagnosis [3]. If doubt persists, a biopsy with histological study provides a conclusive diagnosis.

Although abdominal pseudotumoral tuberculosis is rare, it presents with non-specific and often insidious clinical and radiological features, resembling an abdominal tumor. Diagnostic confirmation may necessitate invasive examinations, such as laparoscopy or laparotomy with biopsy, with only histological confirmation justifying the initiation of anti-tuberculosis treatment [2]. Routine procedures, including specific staining (Ziehl-Neelsen) and culture, are typically performed (with positivity rates ranging from 42% to 69%). Recent reports indicate that bacillus testing by polymerase chain reaction (PCR) on biopsies offers high diagnostic sensitivity (75-80%) and specificity (85-95%) [4]. Treatment relies on anti-tuberculosis drugs, and surgery remains a recourse for compressive or fistulized masses, ensuring definitive resolution of certain cavities [1,3].

## Conclusions

The pseudotumoral manifestation of abdominal tuberculosis exhibits significant diversity in both clinical and radiological expression, occasionally leading to the consideration of alternative diagnoses and mimicking tumoral conditions. The definitive confirmation of this form necessitates histological evidence. This specific case underscores the critical importance of understanding the varied clinical and radiological facets of abdominal tuberculosis, emphasizing the need to consider its possibility in the presence of any lesion resembling a tumor, especially in regions where it is endemic.
